# Destruction of a distal hypoxia response element abolishes *trans*-activation of the *PAG1* gene mediated by HIF-independent chromatin looping

**DOI:** 10.1093/nar/gkv506

**Published:** 2015-05-24

**Authors:** Alexandra Schörg, Sara Santambrogio, James L. Platt, Johannes Schödel, Maja T. Lindenmeyer, Clemens D. Cohen, Katrin Schrödter, David R. Mole, Roland H. Wenger, David Hoogewijs

**Affiliations:** 1 Institute of Physiology and Zürich Center for Integrative Human Physiology ZIHP, University of Zürich, CH-8057 Zürich, Switzerland; 2 Henry Wellcome Building for Molecular Physiology, University of Oxford, Ox3 7BN, UK; 3 Department of Nephrology and Hypertension, Friedrich-Alexander-University Erlangen-Nuremberg, D-91054 Erlangen, Germany; 4 National Center of Competence in Research "Kidney.CH", Switzerland; 5 Institute of Physiology, University of Duisburg-Essen, D-45122 Essen, Germany

## Abstract

A crucial step in the cellular adaptation to oxygen deficiency is the binding of hypoxia-inducible factors (HIFs) to hypoxia response elements (HREs) of oxygen-regulated genes. Genome-wide HIF-1α/2α/β DNA-binding studies revealed that the majority of HREs reside distant to the promoter regions, but the function of these distal HREs has only been marginally studied in the genomic context. We used chromatin immunoprecipitation (ChIP), gene editing (TALEN) and chromosome conformation capture (3C) to localize and functionally characterize a 82 kb upstream HRE that solely drives oxygen-regulated expression of the newly identified HIF target gene *PAG1*. PAG1, a transmembrane adaptor protein involved in Src signalling, was hypoxically induced in various cell lines and mouse tissues. ChIP and reporter gene assays demonstrated that the −82 kb HRE regulates *PAG1*, but not an equally distant gene further upstream, by direct interaction with HIF. Ablation of the consensus HRE motif abolished the hypoxic induction of *PAG1* but not general oxygen signalling. 3C assays revealed that the −82 kb HRE physically associates with the *PAG1* promoter region, independent of HIF-DNA interaction. These results demonstrate a constitutive interaction between the −82 kb HRE and the *PAG1* promoter, suggesting a physiologically important rapid response to hypoxia.

## INTRODUCTION

Hypoxia, defined as mismatch between oxygen supply and consumption, plays a crucial role in many physiological and pathophysiological conditions, such as embryonic development, adaptation to high-altitude, wound healing, inflammation, cardiovascular diseases and cancer. Hypoxia-inducible factors (HIFs) are the master regulators of cellular adaptation to hypoxia (1). HIFs are heterodimeric transcription factors consisting of a labile oxygen-regulated α-subunit and a constitutively expressed β-subunit. Under normoxic conditions, HIFα subunits are hydroxylated by the oxygen-sensing prolyl-4-hydroxylase domain (PHD) enzymes and the factor inhibiting HIF on two prolyl and one asparagine residue, respectively. HIFα prolyl-4-hydroxylation leads to the binding of the E3 ubiquitin ligase von Hippel–Lindau protein (pVHL) followed by polyubiquitination and proteasomal destruction (2,3). Under hypoxic conditions HIFα subunits remain stable, translocate to the nucleus, heterodimerize with HIFβ and transcriptionally activate numerous target genes involved in the adaptation to hypoxia. Therefore, HIF complexes interact directly with the consensus core motif 5′-RCGTG-3′, the actual HIF-binding site (HBS) within the hypoxia response element (HRE) (4).

Pan-genomic studies combining techniques to assess transcriptional activity and protein–DNA interaction functionally identified HREs within the promoter regions as well as HREs far away from transcriptional start sites (TSSs) (5–10). Interestingly, 60% of all HIF-1α and 80% of all HIF-2α interactions with HREs locate to distal sites >2.5 kb outside of the TSS (7). This finding contrasts with the majority of published single-gene studies which show a certain bias (∼76% HREs are within 2.5 kb upstream of the TSSs) toward proximal HREs (4). Furthermore, these pan-genomic analyses also revealed a considerable variability between various cell types, especially for distal HREs (11). Only a few distant HREs have been investigated so far, including those regulating the genes *IGFBP3* (−57 kb upstream of the TSS) (12), *CCND1* (-220 kb) (8), *SLC2A3* (−35 kb) (9) and the putative 5′ kidney-inducible element of the *EPO* gene (−9.2 kb) (13). How these distant HREs interact with the promoter regions, whether this interaction is dependent on the presence of HIFs (or even the HBS itself) and how this promoter–enhancer interaction varies between different cell types remains generally unknown.

By using gene arrays to assess the transcriptional profile of HeLa cells under normoxic and hypoxic conditions, we identified the lipid raft phosphoprotein associated with glycosphingolipid enriched microdomains (*PAG1*) as a novel hypoxia-inducible gene. PAG1 is a ubiquitously expressed transmembrane adaptor protein that binds the protein tyrosine kinase csk and is hence also known as csk-binding protein (14,15). We identified a distal HRE located −82 kb upstream of the *PAG1* TSS and used the TALEN (transcription activator-like effector-based nuclease) technique to specifically target the HBS of this remote HRE, resulting in functional ablation of the hypoxia inducibility of *PAG1* gene transcription. Chromosome conformation capture (3C) assays were employed to assess the interaction between this HRE and the PAG1 promoter region under normoxic, hypoxic, HIF-depleted and HBS-destructed conditions.

## MATERIALS AND METHODS

### Plasmid constructs

A reporter gene plasmid containing 1014 bp of the *PAG1* promoter was obtained from BioCat (Heidelberg, Germany). The *PAG1* −82 kb HRE region was amplified by Phusion polymerase-based polymerase chain reaction (PCR) (Thermo Fisher Scientific, Waltham, MA, USA) using HeLa or MDA-MB-231 genomic DNA and the primers (Microsynth, Balgach, Switzerland) listed in Supplementary Table S1. Following restriction digestion with BglII (Thermo Fisher Scientific) fragments of 317 bp and 2 kb were cloned into the pGL3prom vector (Promega, Madison, WI, USA) upstream of the SV40-driven firefly luciferase gene. The HBS was inactivated by site-directed mutagenesis (5′-CGTG-3′ to 5′-ATAA-3′; Stratagene, La Jolla, CA, USA). All constructs were verified by sequencing (Microsynth). pH3SVL (16), PHD2 (17), PAI-1 and CAIX (18) reporter gene constructs were described previously.

### Cell culture and transfection

HeLa, U2OS, MCF-7, Hep3B, MDA-MB-231, 786–0, 786-VHL, TK188 and TZ-1 cells were cultured in high-glucose Dulbecco's modified Eagle's medium (DMEM) (Sigma, St. Louis, MO, USA). All media were supplemented with 10% heat-inactivated fetal calf serum (FCS) (Gibco-BRL, Grand Island, NY, USA) and antibiotics (50 IU/ml penicillin and 100 μg/ml streptomycin; Sigma). HK2 were cultured in DMEM/F12 (Sigma) supplemented with 10% FCS, 100 IU/ml penicillin, 100 μg/ml streptomycin, 36 ng/ml hydrocortisone and ITS solution (5 μg/ml insulin, 5 μg/ml transferrin, 5 ng/ml selenium; Roche, Basel, Switzerland). Hypoxia experiments were performed at 0.2% or 0.5% O_2_ and 5% CO_2_ in a gas-controlled workstation (InvivO_2_ 400; Baker Ruskinn, Bridgend, South Wales, UK). Cells were transfected using polyethylenimine (Polysciences, Warrington, PA, USA) as described previously (19).

### RNA isolation and analysis

Total RNA was isolated from cultured cells using the guanidine isothiocyanate method as described before (17). RNA from mice exposed to inspiratory hypoxia was obtained as described elsewhere (20). Specific mRNAs were quantified by reverse transcription followed by quantitative PCR (RT-qPCR) using a MX3000P light cycler (Agilent, Santa Clara, CA, USA) as described previously (17). For gene array analysis, total RNA was extracted from HeLa cells cultured under normoxic or hypoxic (16 h, 0.2% O_2_) conditions with RNeasy (Qiagen, Venlo, The Netherlands). RNA integrity was evaluated using the Agilent 2100 Bioanalyzer. Two-colour labelled samples were hybridized to an Agilent whole human genome 4×44K oligonucleotide microarray slide. For each condition two biological replicates were used.

### Analysis of human renal biopsies

Human renal biopsy specimens and Affymetrix microarray expression data (HG-U133 Plus2.0 Array) were procured within the framework of the European Renal cDNA Bank–Kröner-Fresenius Biopsy Bank (21). Diagnostic renal biopsies were obtained from patients after informed consent and with approval of the local ethics committees. Following renal biopsy, the tissue was transferred to RNase inhibitor and microdissected into glomerular (Glom) and tubulointerstitial (Tub) compartments. The microarray expression data used in this study came from individual patients with diabetic nephropathy (DN, Glom (*n* = 7), Tub (*n* = 7), focal segmental glomerulosclerosis (FSGS, Glom (*n* = 16), Tub (*n* = 7)), rapidly progressive glomerulonephritis (RPGN, Glom (*n* = 23), Tub (*n* = 21)) as well as pre-transplant biopsies from living renal allograft donors as controls (LD, Glom (*n* = 18), Tub (*n* = 18)). Total RNA was isolated from microdissected glomeruli and tubulointerstitium, reverse transcribed, and linearly amplified according to a protocol previously reported (22). Fragmentation, hybridization, staining and imaging were performed according to the Affymetrix expression analysis technical manual (Affymetrix, Santa Clara, CA, USA). For microarray analysis Robust Multichip Analysis (RMA) was performed. Following normalized RMA, significance analysis of microarrays was conducted using a q-value of <5% to identify genes that were differently regulated between the analyzed groups (23). RT-qPCR validation of renal biopsies was performed as reported earlier (21). Pre-developed TaqMan reagents were used for human PAG1 (NM_018440.3, Hs00179693_m1) and transcript levels were normalized to 18S rRNA levels (Applied Biosystems, Waltham, MA, USA).

### RNA interference

Expression vectors encoding short hairpin RNA (shRNA) sequences targeting human HIF-1α (Sigma number TRCN0015301048s1c1), HIF-2α (TRCN0014361694s1c1), PAG1 (TRCN00000123272) and a non-targeting control shRNA (shCtrl) under the control of a U6 promoter in a pKLO.1 puromycin resistance vector were purchased from Sigma. Alternatively, the puromycin selection cassette has been replaced by a hygromycin cassette. Lentiviral particles were produced in HEK293T cells using the Vira-Power lentiviral expression vector system according to the manufacturer's instructions (Invitrogen). HeLa cells were infected with lentiviral particles containing shHIF-1α, shHIF-2α or shCtrl RNA, followed by selection with 10 μg/ml puromycin to create shHIF-1α, shHIF-2α and shCtrl cells. To generate shHIF-1α/2α double-knockdown cells, shHIF-2α cells were infected with lentiviral particles containing a hygromycin-resistant shHIF-1α expression vector, followed by selection with 10 μg/ml puromycin and 1 mg/ml hygromycin. Single and double shHIFα MCF-7 as well as shHIF-2α 786–0 cells have been described previously (24–26). Hep3B cells were infected with shPAG1 lentiviral particles followed by selection with puromycin (1.5 μg/ml).

### Protein extraction and analysis

Cells were washed twice with ice-cold phosphate buffered saline (PBS), and soluble proteins were extracted with 10 mM Tris-HCl pH 8.0, 1 mM EDTA, 400 mM NaCl, 0.1% Nonidet P-40, freshly supplemented with 1x protease inhibitor cocktail, 1 mM NaF and 10 mM Na_3_VO_4_ (all obtained from Sigma). Insoluble proteins were further extracted with 1% SDS, 5 mM Tris-HCl pH 6.8, 1% Triton X-100 (Sigma), supplemented as above, by ultrasonication (Cell Disruptor B15, Branson, Danbury, CT, USA). Protein concentration was estimated using the BCA method (Thermo Fisher Scientific) and 60–100 μg total protein were subjected to immunoblot analysis using the following mouse monoclonal (mAb) or polyclonal antibodies (pAb): mAb anti-hHIF-1α (clone 54; BD Transduction Laboratories, San Jose, CA, USA), rabbit pAb anti-hHIF-2α/EPAS1 (Abnova, Taipei City, Taiwan), mAb anti-hPAG1 (clone MEM-255; Abnova). For immunoprecipitation, proteins were extracted using ODG buffer (250 mM Tris-HCl pH 7.4, 1 mM EDTA, 150 mM NaCl, 1% Triton X-100, 60 mM octyl β-d-glucopyranoside, freshly supplemented as above). After pre-clearing, extracted protein (800 μg) was mixed with 8 µg/μl anti-PAG1 mAb (Exbio, Vestec, Czech Republic). Following incubation overnight at 4°C, the resin was washed three times with 1 ml of washing buffer (25 mM Tris-HCl pH 7.4, 150 mM NaCl), resuspended in loading buffer, boiled and subjected to sodium dodecyl sulphate-polyacrylamide gel electrophoresis. Immunoblotting was performed using a pan-tyrosine phosphorylation mAb (Merk Millipore, Darmstadt, Germany) or a rabbit anti-PAG1 pAb (Santa Cruz, Dallas, TX, USA). Primary antibodies were detected with goat-anti-mouse or goat-anti-rabbit HRP-coupled pAb (Thermo Fisher Scientific). Chemiluminescence detection was performed using Supersignal West Dura (Thermo Fisher Scientific) and recorded with a CCD camera (LAS-4000; GE Healthcare, Chalfont, St. Giles, UK) followed by quantification with Quantity One software (Bio-Rad, Hercules, CA, USA). Phosphorylation of 46 specific phosphorylation sites was analysed using the Proteome Profiler Human Phospho-Kinase Array Kit (ARY003B), according to the manufacturer's instructions (R&D Systems, Abingdon, UK). Briefly, Hep3B cells were grown for 24 h, rinsed with ice-cold PBS and resuspended in lysis buffer. Antibody arrays were incubated with cell lysates (350 μg) at 4°C overnight. Signals were detected and quantified as above.

### Reporter gene assays

Cells were co-transfected with 10 ng pRLSV40 *Renilla* control luciferase plasmid (Promega) together with either 3 μg (HeLa) or 400 ng (MCF-7) firefly luciferase plasmid or a combination of each 1.5 μg (HeLa) or 200 ng (MCF-7) firefly luciferase construct and overexpression or empty vectors. After 16 h cells were exposed to either 20% O_2_ or 0.2% O_2_ for the indicated time points. Cells were lysed in 50 μl Passive Lysis Buffer (Promega) and luciferase activities were determined in triplicates using the Dual Luciferase Reporter Assay System according to the manufacturer's protocol (Promega). Reporter gene activities were expressed as ratios between firefly and *Renilla* luciferase activities.

### Gene editing

Pairwise TALEN plasmids to target the −82 kb HBS within the *PAG1* HRE were obtained from Labomics (Nivelles, Belgium). HeLa and MCF-7 cells were transfected with each 1.5 μg TALEN vector and either 500 ng of an EGFP expression vector or 10–30 ng linearised pBabe-puromycin vector. After 16 h, the cells transfected with EGFP were cloned by limited dilution in 96-well plates and EGFP expressing cells were further expanded. After 72 h, the puromycin co-transfected cells were selected with 10 μg/ml (HeLa) or 2 μg/ml (MCF-7) puromycin. Resistant cells were cloned by limited dilution in 96-well plates. Following expansion of the cloned cells, genomic DNA was isolated and the −82 kb *PAG1* region amplified by PCR as described above. PCR products were either digested with BsaAI (New England Biolabs, Ipswich, MA, USA) and analyzed by 1% NuSieve agarose gel electrophoresis (Lonza, Basel, Switzerland) or cloned into a plasmid vector and sequenced (Microsynth).

### Chromatin immunoprecipitation and chromosome conformation capture (3C)

Chromatin immunoprecipitation (ChIP) experiments using HIF-1α, HIF-2α, HIFβ, histone modifications or p300 occupancy were performed as described previously (7,8). Samples were analysed by qPCR using the primers listed in Supplementary Table S2. All values were displayed as fold enrichment over the negative control from three independent experiments. 3C experiments were performed as described (27) with some modifications. Cells were grown under normoxic or hypoxic (0.2% O_2_, 24 h) conditions and cross-linked with ethylene glycol-bis(succinimidyl succinate) (Thermo Fisher Scientific) for 45 min at room temperature. Nuclei were fixed with 1% formaldehyde for 10 min, quenched with 1% glycine and digested with EcoRI. Fragments were diluted in ligation buffer and ligation was performed for 4 h at 16°C using T4 ligase. Cross-linking was reversed at 65°C overnight and DNA was extensively purified before PCR amplification using the primers listed in Supplementary Table S3. An equimolar mixture of two BAC clones (RP11–624P8 and RP11–21K19), covering the entire region of interest, was used to create an artificial library of ligation product to control for PCR efficiency. The *EEF1G* locus was used to control for cross-linking efficiency between different experiments. PCR products were run on agarose gels, recorded by AlphaImager (Alpha Innotech) and quantified with ImageQuant software. All data were normalized to the BAC ligation products and the *EEF1G* control.

### Statistical analysis

If not otherwise indicated, results are presented as mean values ± standard error of the mean (SEM) of at least three independent experiments. Statistical analyses were performed using GraphPad Prism version 4.0 (GraphPad Software).

## RESULTS

### Hypoxia induces PAG1 expression

Gene array analysis of HeLa cells cultured under normoxic or hypoxic conditions revealed a number of previously unreported oxygen-regulated genes (Supplementary Table S4). mRNA levels of several well-established HIF target genes, including *CA9, NDRG1, LOX* and *EGLN3*, were also upregulated, confirming the reliability of this transcriptome analysis. One of the novel, previously unpublished genes was *PAG1*, a transmembrane adaptor protein known to interfere with Src signalling (14,15). PAG1 attracted our attention because it had been reported to be overexpressed in clear cell renal cell carcinoma (ccRCC) which is frequently associated with loss of VHL function and constitutive HIF-2α overexpression (28). Furthermore, *PAG1* was among the high stringency genes with remote HIF-2 binding sites (7), suggesting that it may serve as prototype gene to study gene activation by remote HIF-2-dependent HREs.

**Figure 1.**
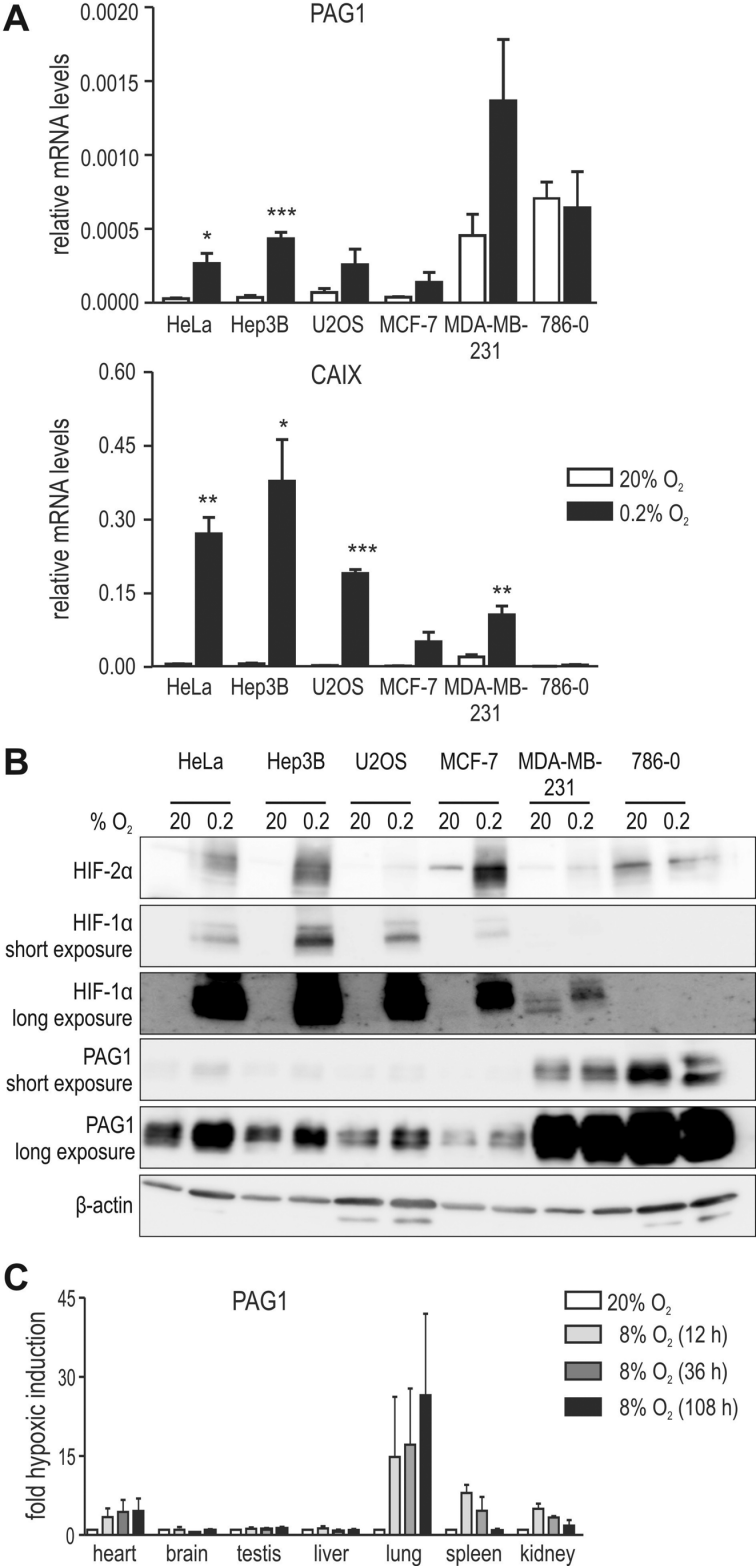
Oxygen-regulated PAG1 expression *in vitro* and *in vivo*. (**A**) The indicated cancer cell lines were exposed to 20% or 0.2% O_2_ for 24 h and mRNA levels of PAG1 (top panel) and CAIX (bottom panel) were determined by RT-qPCR. Results of at least three independent experiments are shown relative to β-actin control mRNA levels. Error bars correspond to the SEM and statistical analyses were performed with an unpaired Student's t-test (*P < 0.05; **P < 0.01; ***P < 0.001). (**B**) Cells were treated as in (A) and protein levels of HIF-2α, HIF-1α, PAG1 and β-actin were determined by immunoblotting. (**C**) Groups of three C57Bl/6 mice were exposed to inspiratory hypoxia (8% O_2_) for 12–108 h. PAG1 mRNA levels in the organs indicated were determined by RT-qPCR and normalised to ribosomal protein S12 mRNA levels. Results are displayed as fold hypoxic induction, error bars correspond to the SEM.

We first confirmed hypoxic PAG1 induction in a panel of cancer cell lines derived from a broad range of different tissues. mRNA levels of both PAG1 and the well-known hypoxia-inducible CAIX were robustly upregulated by 3- to 11-fold in all cancer cell lines examined, except in VHL-deficient 786–0 ccRCC in which PAG1 levels were constitutively high (Figure 1A). Similar results were obtained on the protein level (Figure 1B). To analyze hypoxic induction of PAG1 *in vivo*, we quantified mRNA levels in various tissue samples derived from mice exposed to inspiratory hypoxia (8% O_2_) for up to 108 h. A profound time-dependent PAG1 upregulation could be observed in heart, lung, spleen and kidney with maximal induction factors ranging from 2- to 7-fold (Figure 1C).

Regarding the hypoxic PAG1 induction in mouse kidney, we next analyzed gene array data from human renal biopsy specimens collected from the European Renal cDNA Bank (21). Data were obtained from various nephropathies, including FSGS, RPGN, DN as well as pre-transplant biopsies from living renal allograft donors as controls. Emerging evidence suggests that dysregulation of hypoxia-regulated transcriptional mechanisms contributes to the loss of renal function and the development of chronic kidney disease. A consistent and significant association of elevated PAG1 levels with advanced end-stage kidney disease was found for the glomerular as well as tubulointerstitial compartment (Supplementary Figure S1A). These gene array data were validated by RT-qPCR in an independent patient cohort, supporting the association of induced PAG1 levels and several advanced glomerulopathies (Supplementary Figure S1B). Hypoxically elevated PAG1 mRNA levels were also found in immortalized human cell lines derived from different renal compartments, including HK2 proximal tubular cells (Supplementary Figure S1C) and TK188 and TZ-1 renal fibroblasts (Supplementary Figure S1D). In summary, these data suggest that PAG1 is hypoxically induced under various physiological as well as pathological conditions affecting renal function.

### PAG1-mediated basal regulation of Src signalling is not altered by hypoxic conditions

PAG1 has been reported to be involved in the regulation of Src family kinases (SFKs) (14,15). To address the potential functional consequences of PAG1 hypoxic regulation we used Hep3B cells, which contain a combination of relatively high basal and strong hypoxia-inducible PAG1 levels, and generated shPAG1 Hep3B cells. PAG1 knockdown efficiency was validated on the mRNA and protein levels (Supplementary Figure S2A). A proteome profiler phospho-kinase array screen did not reveal any PAG1-dependent difference in SFK activity, in shPAG1 compared with shCtrl Hep3B cells under normoxic or hypoxic conditions (Supplementary Figure S2B). Consistently, following PAG1 immunoprecipitation and detection with a pan-phosphotyrosine antibody, no change in specific PAG1 phosphorylation could be observed while total PAG1 protein levels were again increased (Supplementary Figure S2C).

### *PAG1* is a HIF target gene

**Figure 2.**
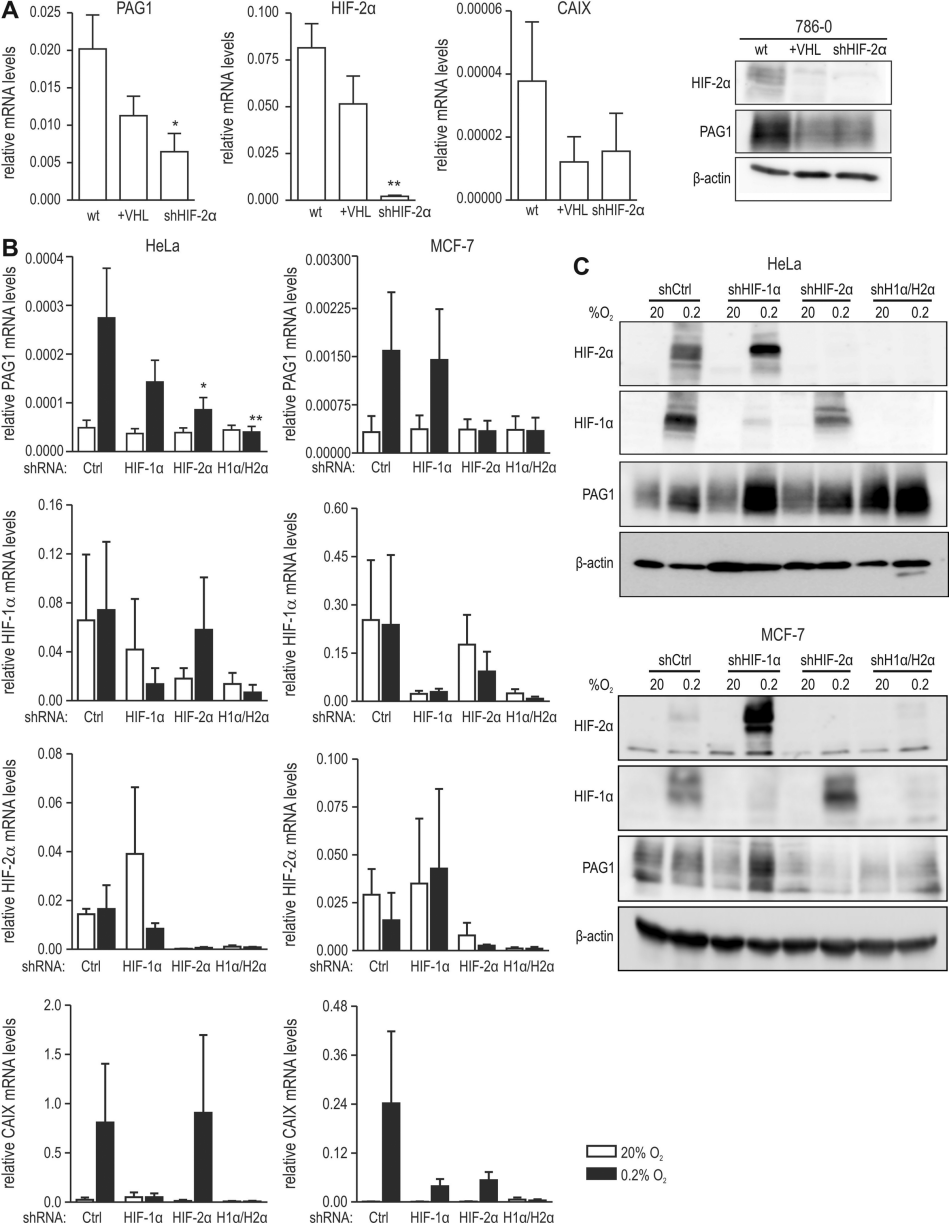
HIF-dependent PAG1 expression. (**A**, left panel) PAG1 mRNA levels in VHL-deficient 786–0, VHL-reconstituted 786-VHL and shHIF-2α 786–0 ccRCC cells. HIF-2α and CAIX mRNA levels were included as controls. All mRNA expression levels were determined by RT-qPCR and displayed relative to the β-actin mRNA levels (*n* = 3), error bars correspond to the SEM. Statistical analyses were performed with one-way ANOVA and Dunnet's correction for multiple comparisons (*P < 0.05; **P < 0.01). (A, right panel) Protein levels of HIF-2α, PAG1 and β-actin were determined by immunoblotting of 786–0 cells. (**B**) HeLa and MCF-7 cells were stably transfected with shCtrl, shHIF-1α and/or shHIF-2α, and exposed for 24 h to 20% O_2_ or 0.2% O_2_. PAG1, CAIX, HIF-1α and HIF-2α mRNA levels were quantified by RT-qPCR and shown relative to β-actin mRNA levels (*n* = 3), error bars correspond to the SEM. Statistical analyses were performed with one-way ANOVA and Dunnet's correction for multiple comparisons (*P < 0.05; **P < 0.01). (**C**) HeLa and MCF-7 cells were treated as in (B) and HIF-2α, HIF-1α, PAG1 and β-actin proteins detected by immunoblotting.

The abundant and oxygen-independent PAG1 levels in 786–0 cells suggested PAG1 regulation by the VHL/HIF-2α axis. Indeed, both reconstitution of VHL as well as shRNA-mediated knockdown of HIF-2α not only reduced HIF-2α but also PAG1 mRNA and protein levels (Figure 2A).

To investigate a potential transcriptional selectivity for a specific HIFα isoform, PAG1 transcript levels were analysed in shHIF-1α and/or shHIF-2α HeLa and MCF-7 cell lines cultured under normoxic and hypoxic conditions. Hypoxic PAG1 mRNA induction was significantly attenuated by shHIF-2α in HeLa cells and entirely eliminated by shHIF-2α in MCF-7 cells (Figure 2B). HIF-1α shRNA had no significant effect on hypoxic PAG1 expression in MCF-7 cells and only a minor effect in HeLa cells. In both cell lines, combined HIF-1α/HIF-2α knockdown abolished hypoxic PAG1 induction (Figure 2B). Similar results were obtained for PAG1 protein levels (Figure 2C). Consistent with our previous findings (24,29), increased HIF-2α could be observed in the absence of HIF-1α, which further induced hypoxic PAG1 protein levels (Figure 2C). These data demonstrate that at least in the cell lines analyzed PAG1 is predominantly regulated by HIF-2α.

### Localization of a putative distal HRE −82 kb upstream of the *PAG1* TSS

**Figure 3.**
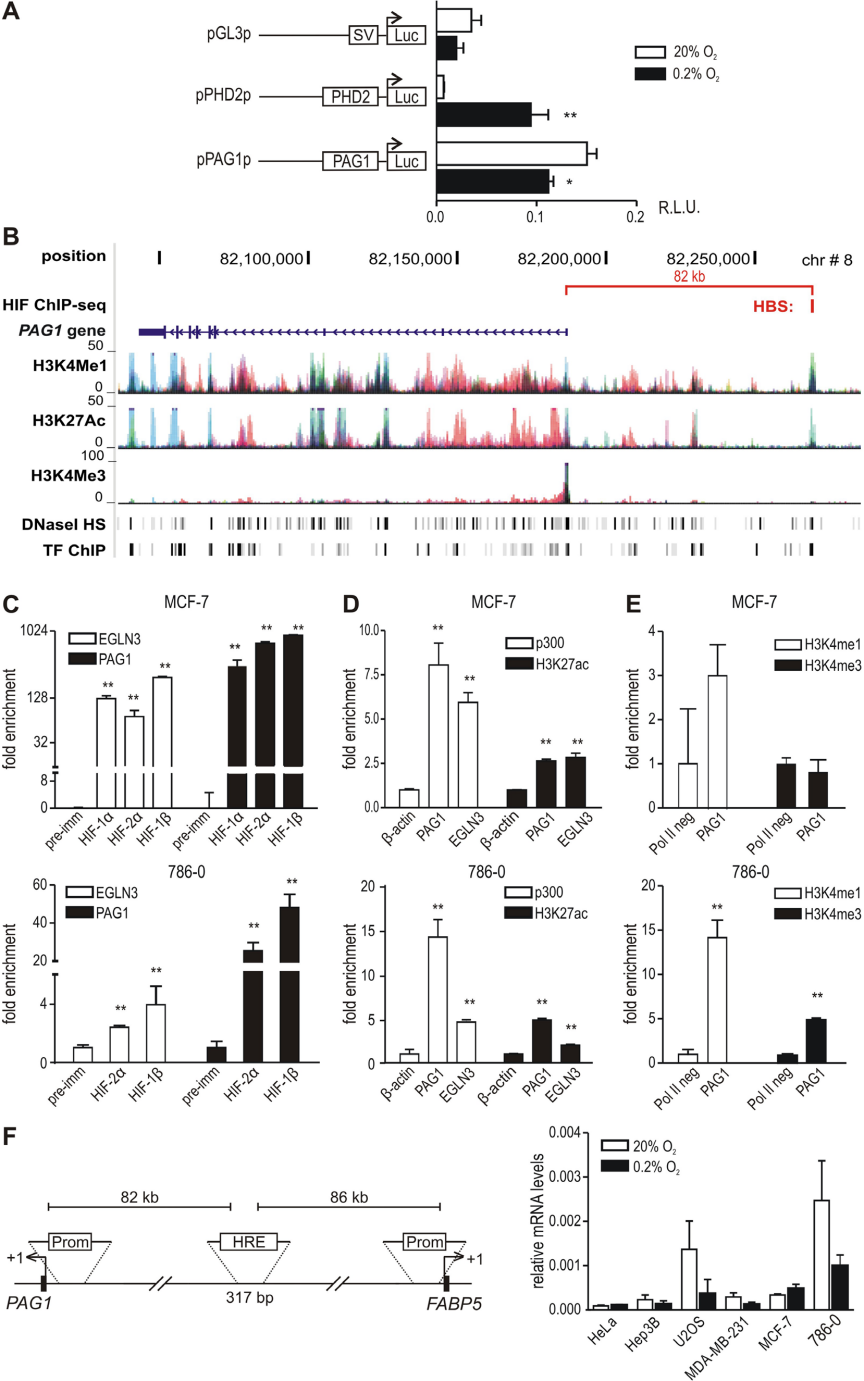
Identification of a HRE 82 kb upstream of the *PAG1* TSS. (**A**) A *PAG1* promoter-driven firefly luciferase reporter gene was transiently co-transfected into HeLa cells followed by exposure to 20% O_2_ or 0.2% O_2_ for 24 h. An SV40 promoter and a *PHD2* promoter-driven luciferase vector served as negative and positive controls, respectively. Results are displayed as ratios of firefly to *Renilla* luciferase activities in relative light units (R.L.U.) from three independent experiments performed in triplicates, error bars correspond to the SEM. Statistical analyses were performed with unpaired Student's t-tests (*P < 0.05; **P < 0.01). (**B**) UCSC Genome Browser output *(hg18)* of the *PAG1* genomic region, illustrating the location of the putative −82 kb HRE. In addition, the ENCODE integrated regulation track containing H3K4Me1/3 marks, H3K27Ac marks, DNaseI hypersensitivity clusters and transcription factor ChIP-seq data are displayed. Colors in the histone mark tracks represent conventional ENCODE codes used to illustrate different cell lines. (**C**-**E**) ChIP-qPCR of normoxic 786–0 cells and hypoxic (0.5% O_2_, 16 h) MCF-7 cells using antibodies directed against HIF-1α, HIF-2α, HIFβ, p300 and histone modifications H3K27Ac, H3K4Me1 and H3K4Me3. The HRE of *EGLN3/PHD3*, located in the first intron, served as positive control. Statistical analyses were performed with one-way ANOVA and Dunnet's correction for multiple comparisons (*P < 0.05; **P < 0.01). (**F**, left panel) Scheme depicting the location of the putative −82 kb HRE between the TSSs of *PAG1* and *FABP5*. (F, right panel) The indicated cell lines were exposed to 20% O_2_ or 0.2% O_2_ for 24 h and FABP5 mRNA levels were quantified by RT-qPCR and shown relative to β-actin mRNA levels (*n* = 3).

In order to assess the molecular mechanism of HIF-mediated *PAG1* transactivation, we first analyzed a 1 kb promoter fragment upstream of the *PAG1* TSS, containing a single consensus HRE motif, in a reporter gene assay. However, in contrast to a similar construct driven by the established hypoxia-inducible PHD2 promoter (17), the PAG1 promoter was not induced by hypoxia (Figure 3A).

We next interrogated the genome-wide HBS ChIP-seq datasets (7) and identified an intergenic HIF-2α binding site 82 kb upstream of the *PAG1* TSS in both MCF-7 and 786–0 cells (Supplementary Figure S3). This site contains a consensus HRE motif, overlaps with DNaseI hypersensitivity clusters that reflect open chromatin and displays several epigenetic marks of active enhancers as indicated by the UCSC-integrated ENCODE data (Figure 3B). Furthermore, the remote site contains strong transcription co-factor occupancy, including p300 and RNApol2, as illustrated by the compressed transcription factor ChIP-seq ENCODE track in Figure 3B and expanded view in Supplementary Figure S3. During the course of our study this distant locus was reported as a HIF-1 binding site detected by ChIP-seq in HUVEC cells (9). ChIP-qPCR experiments for HIF-1α, HIF-2α and HIFβ in MCF-7 and 786–0 cells independently validated a robust binding of all three HIF subunits at this site (Figure 3C). Common binding of HIF-1 and HIF-2 is frequently observed at loci that are transcriptionally regulated by a single isoform, reflecting post-binding mechanisms of transcriptional selectivity (7). Consistent with the ENCODE data, ChIP-qPCR-based analysis of histone marks revealed high levels of H3K27Ac and H3K4Me1, with low levels of H3K4Me3, in MCF-7 and 786–0 cells (Figure 3D and E). This combination of histone modifications is usually observed at active enhancers but not promoters. These results together with the lack of detection of any transcript in this region using MCF-7-based RNA-sequencing (30) exclude that this site is regulating a potential proximal unannotated transcript. Furthermore, substantial enrichment of this locus was observed upon transcriptional co-activator p300 precipitation (Figure 3D). p300 is a well-known essential transcriptional co-activator of HIFs (31).

Of note, the TSS of *FABP5* (fatty acid binding protein 5) is located 86 kb further upstream of the putative *PAG1* HRE (indicated in Figure 3F, left part), suggesting that *FABP5* might also be oxygen-regulated. However, in line with a previously reported study (32), RT-qPCR quantification of *FABP5* transcript levels did not reveal any hypoxic induction in the cell lines used above (Figure 3D, right part). Moreover, physical association experiments exclude an interaction of the intergenic −82 kb HRE with the *FABP5* promoter (J. Platt and D. Mole, unpublished results). Collectively, these data indicate that the remote −82 kb HRE might be involved in the transcriptional regulation of *PAG1* but not the equally distant *FABP5* gene.

### Hypoxic activation of reporter gene expression by the −82 kb *PAG1* HRE

**Figure 4.**
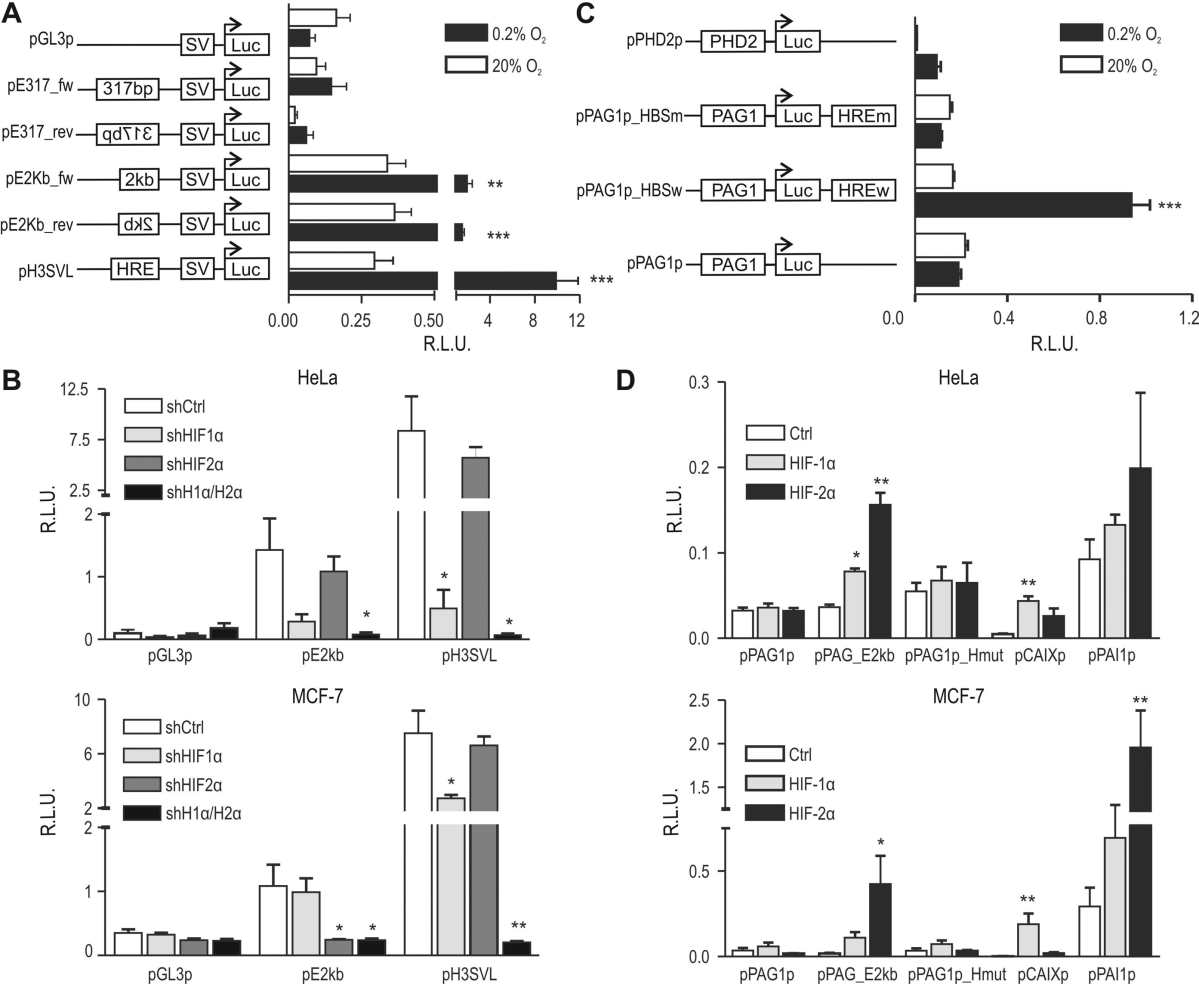
Hypoxic *cis*-activation of reporter genes by the −82 kb *PAG1* HRE. (**A**) A 317 bp or a 2 kb fragment, both including the −82 kb *PAG1* HRE, was used to enhance expression of an SV40 promoter-driven firefly luciferase reporter gene vector (pGL3p). pH3SVL, containing three HREs with tandem HBSs derived from the *transferrin* gene, was used as a positive control. Following transient co-transfection, HeLa cells were exposed to 20% O_2_ or 0.2% O_2_ for 24 h. Results are displayed as ratios of firefly to SV40 promoter-driven *Renilla* luciferase activities in relative light units (R.L.U.) from three independent experiments performed in triplicates, error bars correspond to the SEM. Statistical analyses were performed with unpaired Student's t-tests (**P < 0.01; ***P < 0.001). (**B**) HeLa and MCF-7 cells, stably transfected with shCtrl, shHIF-1α and/or shHIF-2α, were additionally transiently transfected with the indicated reporter genes. Statistical analyses were performed with one-way ANOVA and Dunnet's correction for multiple comparisons (*P < 0.05; **P < 0.01). (**C**) The 2 kb HRE fragment, containing the wild-type (HBSw) or a mutant HBS (HREm), was used to enhance expression of a *PAG1* promoter-driven firefly luciferase reporter gene (pPAG1p). A *PHD2* promoter-driven luciferase vector served as positive control. Statistical analyses were performed with unpaired Student's t-test (***P < 0.001). (**D**) HeLa and MCF-7 cells were transiently co-transfected with the indicated reporter gene vectors together with empty, HIF-1α and HIF-2α overexpression vectors. The *CA9* and *PAI1* promoter-driven luciferase vectors served as HIF-1α and HIF-2α, respectively, isoform-specific controls. Statistical analyses were performed with one-way ANOVA and Dunnet's correction for multiple comparisons (*P < 0.05; **P < 0.01). (B–D) Exposure to hypoxia and determination of reporter gene activity was performed as in (A).

To evaluate the functionality of the putative −82 kb HRE, heterologous reporter gene assays were performed using a 317 bp fragment encompassing the HBS and covering the entire high stringency HIF-2 binding site (7). This 317 bp fragment was cloned in both orientations upstream of a heterologous SV40 promoter (Figure 4A). pH3SVL, an SV40 promoter-driven luciferase reporter gene containing three concatamerized HREs derived from the −3.5 kb *transferrin* enhancer (33), was used as positive control. In contrast to pH3SVL, the 317 bp fragment did not significantly increase firefly luciferase activity in hypoxic HeLa cells (Figure 4A). However, a 2 kb fragment of the same −82 kb region significantly enhanced hypoxic reporter gene induction, regardless of its orientation (Figure 4A), confirming that the minimal HBS is not sufficient to constitute a functional HRE but rather includes additional *cis*-acting elements. Indeed, only the 2 kb fragment covers the entire ENCODE-derived H3K4Me1 and H3K27Ac tracks found in the UCSC Genome Browser (Supplementary Figure S3).

To assess HIFα isoform-specific hypoxic *trans*-activation of the 2 kb fragment encompassing the −82 kb *PAG1* HRE, single or double shHIFα-mediated knockdown cells were transfected with reporter gene constructs. Consistent with endogenous PAG1 protein regulation (see above), shHIF-1α/shHIF-2α double-knockdown HeLa and MCF-7 cells were not able to induce HRE-dependent luciferase activity (Figure 4B). *PAG1* −82 kb HRE-driven reporter gene activity was mainly impaired by shHIF-1α single-knockdown in HeLa cells and shHIF-2α single-knockdown in MCF-7 cells (Figure 4B), as observed for endogenous PAG1 expression shown above.

To explore the functional interaction of the *PAG1* promoter with the −82 kb HRE, luciferase reporter genes were constructed containing the 1 kb promoter fragment 5′ of the luciferase gene and the 2 kb HRE fragment 3′ to the luciferase gene to mimic its distal location on the circular plasmid (Figure 4C). An established *PHD2* promoter-driven reporter gene was included as positive control (17). As observed before, the *PAG1* promoter alone conferred basal but not hypoxically induced reporter gene expression. However, the −82 kb *PAG1* HRE significantly enhanced *PAG1* promoter activity under hypoxic conditions (Figure 4C). Mutation of the −82 kb HRE (5′-CGTG-3′ to 5′-ATAA-3′) completely abrogated hypoxic reporter gene induction (Figure 4C).

Finally, overexpression of hydroxylation-resistant HIFα isoforms in HeLa and MCF-7 cells confirmed HIF-mediated activation of reporter gene expression under the control of the −82 kb *PAG1* HRE (Figure 4D). The promoters derived from the genes encoding PAI-1 and CAIX served as controls for hypoxic induction preferentially driven by HIF-2 and HIF-1, respectively. Notably, overexpressed HIF-2α enhanced *PAG1* promoter-driven reporter gene expression substantially better than HIF-1α in both cell lines, and mutation of the −82 kb HRE again fully abrogated HIF responsiveness of the reporter gene (Figure 4D).

### Destruction of the −82 kb *PAG1* HRE abrogates hypoxic PAG1 mRNA induction

**Figure 5.**
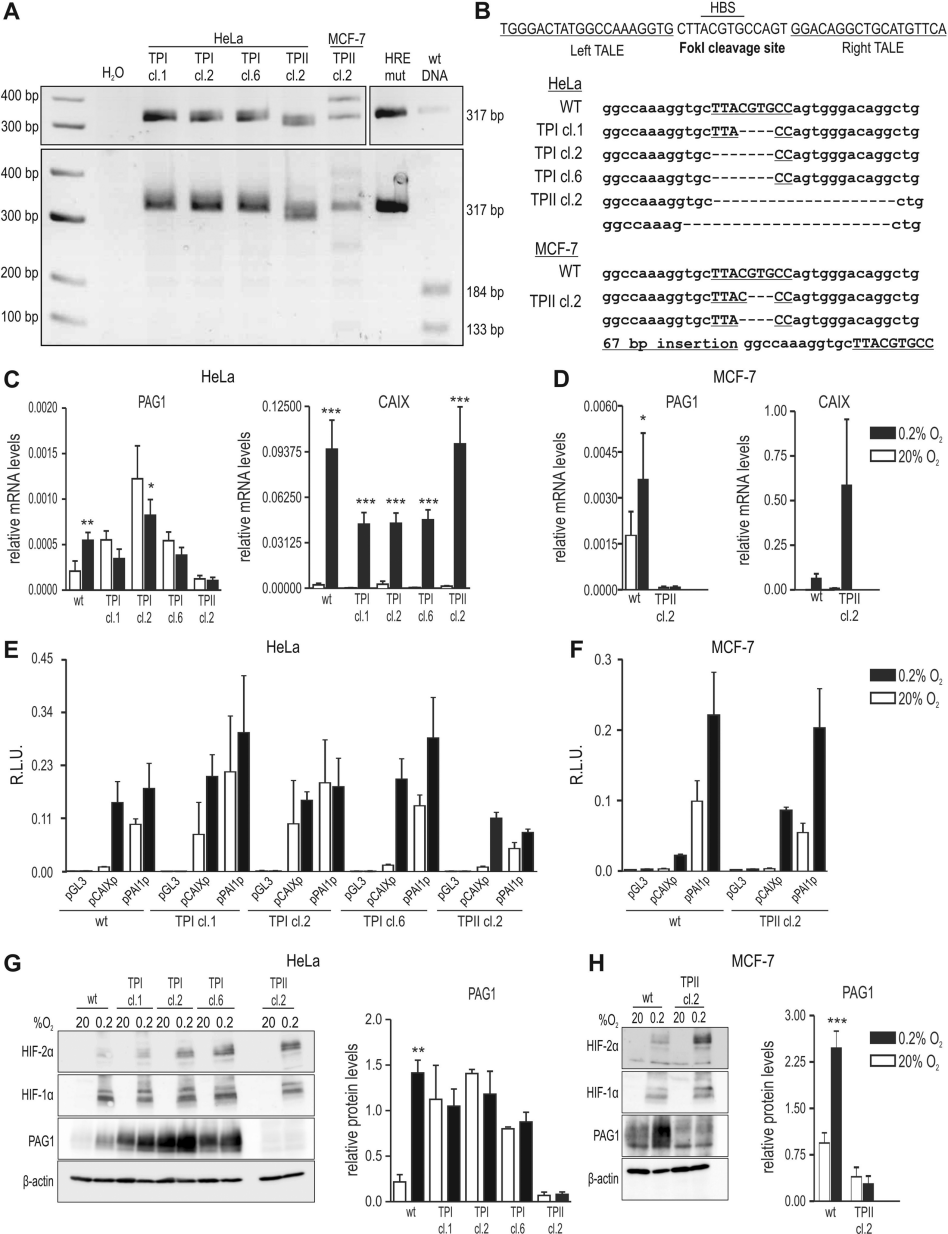
TALEN-mediated destruction of the −82 kb *PAG1* HRE. (**A**) PCR analysis of representative HeLa and MCF-7 clones following co-transfection of two independent TALEN pairs (TPI and TPII) targeting the HBS within the −82 kb *PAG1* HRE. A 317 bp fragment encompassing the putative HBS was amplified by PCR and restriction digested with (lower panel) or without BsaAI (upper panel). (**B**) DNA sequence analysis of the cell clones shown in (A). Dashes indicate deleted bases. Wild-type (wt) and targeted HeLa (**C**) and MCF-7 (**D**) clones were exposed to 20% O_2_ or 0.2% O_2_ for 24 h, followed by quantification of PAG1 and CAIX mRNA levels. Results of three independent experiments are shown relative to the β-actin control mRNA levels. Statistical analyses were performed with unpaired Student's t-tests (*P < 0.05; **P < 0.01). Targeted HeLa (**E**) and MCF-7 (**F**) clones were transiently transfected with the indicated reporter gene vectors. Exposure to hypoxia and determination of reporter gene activity was performed as described in Figure 4A. Proteins from HeLa (**G**) and MCF-7 (**H**) cultures treated as above were analysed for HIF-2α, HIF-1α, PAG1 and β-actin levels by immunoblotting (left panels). A single representative immunoblot is shown. Following quantification of three independent immunoblots, PAG1 band intensities were shown relative to the intensities of the β-actin bands (right panels). Results are displayed as mean ± SD of *n* = 3. Statistical analyses were performed with unpaired Student's t-tests (**P < 0.01; ***P < 0.001).

Because in total 72 canonical 5′-RCGTG-3′ HBS sequence motifs are located within the 82 kb region upstream of the *PAG1* TSS, it is well possible that additional functional HREs might also mediate hypoxic PAG1 induction. To analyze whether the −82 kb *PAG1* HRE is necessary for PAG1 regulation, we used the TALEN technique (34,35) to disrupt the −82 kb HRE in HeLa and MCF-7 cells. Two different pairs of TALEN targeting vectors (referred to as TPI and TPII) were constructed (Supplementary Figure S4A). Following co-transfection with TPI or TPII, HeLa and MCF-7 cells, either positive for EGFP fluorescence or puromycin resistance, were sub-cloned and the −82 kb HRE amplified by PCR. BsaAI resistant PCR products indicated destruction of the HBS within the −82 kb HRE due to *FokI* endonuclease cleavage followed by non-homologous end joining (NHEJ) DNA repair (Supplementary Figure S4B). Of 96 cell clones that were genotyped, 24 were found to be BsaAI resistant on either one or both alleles (Figure 5A, Supplementary Figure S4C). DNA sequence analysis revealed different NHEJ-mediated repair, demonstrating independent clones (Figure 5B).

*PAG1* −82 kb HRE wild-type and mutant cell clones were cultured under normoxic or hypoxic conditions and PAG1 mRNA levels quantified. Whereas wild-type cells consistently displayed significant oxygen-dependent PAG1 expression levels, hypoxic inducibility of PAG1 mRNA was lost in all HeLa (Figure 5C, left part) and MCF-7 (Figure 5D, left part) cell clones analyzed, demonstrating that this distant HRE is solely responsible for hypoxic PAG1 regulation. In contrast, hypoxic CAIX mRNA induction remained unaffected (Figure 5C and D, right part), suggesting that the oxygen-sensing pathway is still intact in these newly generated sub-clones of the HeLa and MCF-7 cell lines. Consistently, EGFP positive or puromycin resistant clones which contained two wild-type or only one targeted allele still showed hypoxic PAG1 (and CAIX) mRNA induction (Supplementary Figure S4D and E). For HeLa clone 13 this apparent hypoxic induction was however not significant (Supplementary Figure S4D).

To further rule out any potential TALEN off-target effects, we performed reporter gene assays using the HIF-1-dependent *CA9* and HIF-2-dependent *PAI1* promoters to drive firefly luciferase gene expression in *PAG1* −82 kb HRE mutant HeLa and MCF-7 cells. Although some non-significant variability could be observed, HIF-1 and HIF-2 driven reporter gene activity was similar if not even more pronounced in these cells (Figure 5E and F), confirming that general changes in the HIF oxygen sensing pathway did not account for the TALEN-mediated loss of hypoxic PAG1 induction upon mutation of the −82 kb HRE.

The data obtained on PAG1 mRNA levels were confirmed by loss of hypoxic PAG1 protein induction upon −82 kb HRE mutation in HeLa (Figure 5G, Supplementary Figure S4F) and MCF-7 cells (Figure 5H, Supplementary Figure S4G). Quantification of protein levels again displayed significant hypoxically induced PAG1 expression levels in wild-type cells, which was absent in the mutant cells (Figure 5G and H, right panel). Unexpectedly, some of the targeted HeLa cell clones displayed increased basal PAG1 mRNA (Figure 5C, Supplementary Figure S4D) and protein (Figure 5G, Supplementary Figure S4F) levels, similar to the double shHIF-1α/shHIF-2α HeLa cells (Figure 2C). While we currently have no explanation for these clonal changes in PAG1 expression, they did not affect the conclusions regarding the function of the −82 kb HRE drawn from these experiments.

Whereas our TALEN data conclusively confirmed the functionality of the −82 kb HRE, comprehensive ENCODE analysis provided additional support for the functionality of the distal HRE in comparison to all other non-functional HREs in the upstream PAG1 region: only three of the 72 5′-RCGTG-3′ motifs overlap with DNaseI hypersensitivity sites in >10 out of 125 cell lines analyzed (Version 3 in *hg19*), consistent with the earlier hypothesis that DNaseI hypersensitivity in normoxic cells represents an important predictor of HIF binding to its consensus recognition site. Indeed, it has been demonstrated previously that DNaseI hypersensitive 5′-RCGTG-3′ motifs are 19 times more likely to bind HIF-1 and 22 times more likely to bind HIF-2 compared with DNaseI insensitive motifs (7). Importantly, none of the potential PAG1 upstream HRE sites, with exception of the promoter, contains a similar high transcription factor occupancy as the −82 kb HRE and none of the 72 5′-RCGTG-3′ motifs coincides with a unique combination of histone mark H3K4Me1, strong transcription factor occupancy and robust DNaseI hypersensitivity (Supplemental Figure S3).

### Chromatin interaction between the *PAG1* promoter and the −82 kb HRE enhancer is independent of HIF

**Figure 6.**
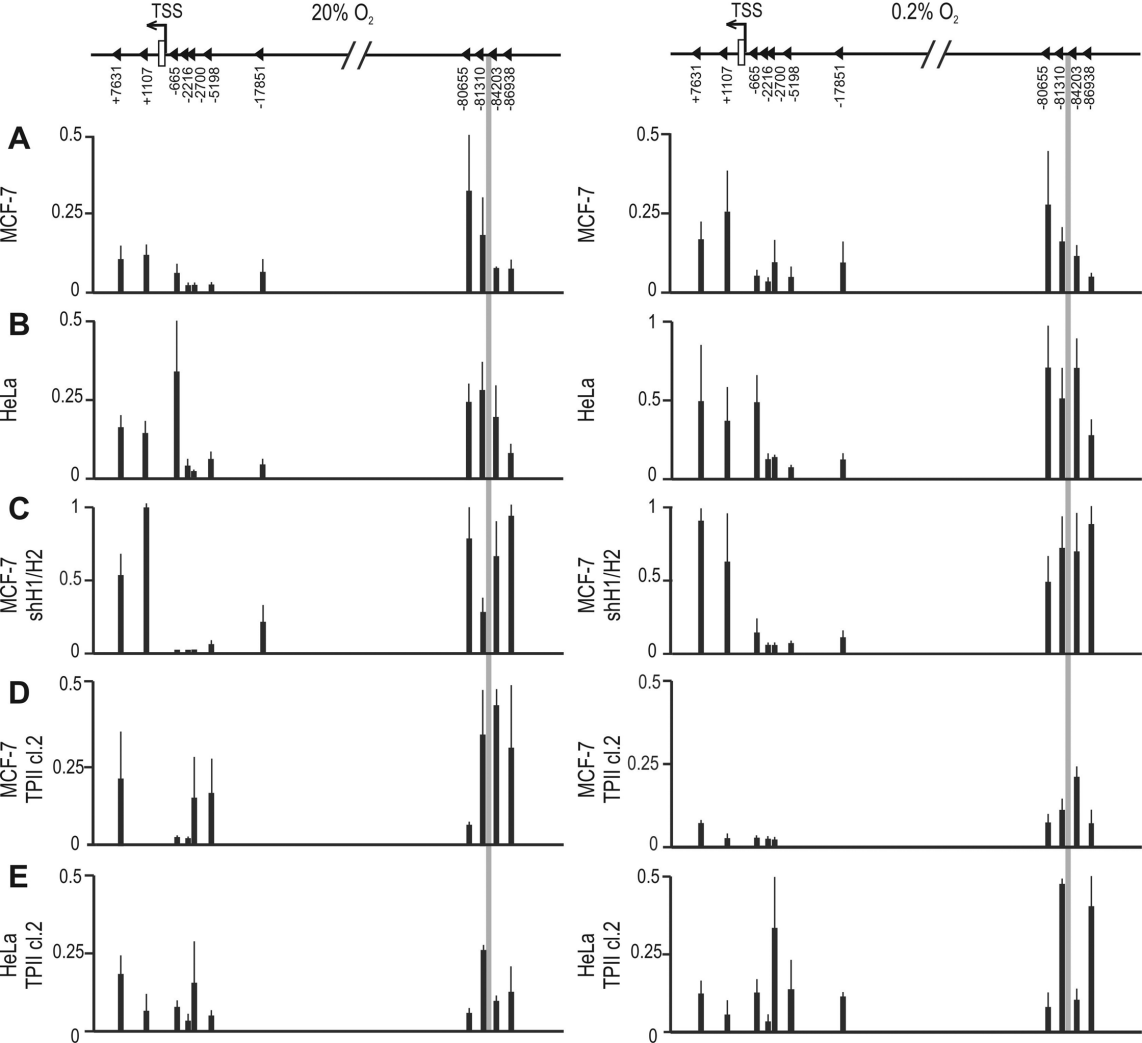
3C assays of a 95 kb region of the *PAG1* gene comprising the first intron to 4.9 kb upstream of the −82 kb HRE, as schematically indicated. The grey bar represents the location of the anchor fragment containing the −82151 bp HRE, triangles correspond to primer locations adjacent to the EcoRI sites. MCF-7 and HeLa wild-type (**A** and **B**, respectively), shHIF-1α/2α MCF-7 (**C**), and MCF-7 and HeLa HBS-destructed (**D** and **E**, respectively) cells were exposed to normoxic (left panel) or hypoxic (right panel) conditions for 24 h. Relative interaction frequencies were obtained in triplicate from three independent experiments, error bars correspond to the SEM.

To investigate the physical interaction between the −82 kb HRE with the *PAG1* promoter region, 3C assays were performed using the −82 kb enhancer as bait. Following cross-linking and EcoRI cleavage, re-ligation products were analyzed by unidirectional semi-quantitative PCR on agarose gels as indicated by the scheme shown on top of Figure 6. This method allowed the exclusion of incomplete EcoRI cleavage as a potential reason for fragment proximity. EcoRI digested and re-ligated BAC clones, covering the entire genomic region investigated, served as PCR efficiency controls, and the *EEF1G* locus was used to ensure equal cross-linking efficiency between the experiments.

PCR products were generally observed in MCF-7 (Figure 6A) and HeLa (Figure 6B) cells between the −83637 bp anchor primer and the +7631 bp as well as +1107 bp primers, both located in the first *PAG1* intron, suggesting that the −82 kb HRE directly interacts with the TSS of *PAG1*. Consistently, both intronic sites coincide with a DNaseI hypersensitivity signal in MCF-7, as deduced from publically available ENCODE data. These intronic interactions also largely corroborate with recent genome-wide RNApol2 binding studies showing that for the majority of hypoxia-inducible genes, RNApol2 is already bound at the promoter in normoxic cells and hypoxia does not substantially increase promoter-bound RNApol2, but rather leads to an increase in RNApol2 across the body of the gene (30), suggesting that HIF is involved in elongation of transcription rather than transcription initiation complex formation. In HeLa cells, an additional strong interaction could be observed between the distal enhancer and the most proximal promoter fragment (Figure 6B).

Interestingly, hypoxic exposure (0.2% O_2_, 24 h) of MCF-7 and HeLa cells did not significantly alter the distal chromatin interaction. We also detected considerable proximal chromatin interactions in both cell lines under normoxic as well as hypoxic conditions.

The lack of significant hypoxic changes in chromatin looping suggested that HIF is not involved in these interactions. Indeed, in shHIF-1α/2α MCF-7 cells (see Figure 2C) a similar, even more pronounced general pattern of chromatin looping could be observed (Figure 6C), providing further evidence that induction of HIF is not required for chromatin interactions with the −82 kb HRE.

Finally, we repeated the 3C experiments in MCF-7 and HeLa cells following TALEN-mediated destruction of the HBS within the −82 kb *PAG1* HRE. Chromatin interactions with the mutant HBS-containing EcoRI fragment generally became weaker in MCF-7 (Figure 6D) and HeLa (Figure 6E) cells when compared to the maternal cell lines (Figure 6A and B). However, while most of the local interactions still appeared more robust than the background interaction frequencies of the HBS region with the EcoRI fragments in between the *PAG1* promoter and HRE regions, the distal interactions with the promoter region did not exceed anymore those background levels. Of note, whereas distal looping was indistinguishable from basal interactions, several local loops remained, suggesting that at least some of the proximal loops required an intact HRE and did not merely occur by chance due to proximity.

## DISCUSSION

In this paper, we report on the oxygen-dependent regulation of the *PAG1* gene which we newly identified by gene array analysis of hypoxic HeLa cells. PAG1 mRNA levels were robustly induced in various human cell lines and mouse tissues. This finding is quite remarkable because in tissues *in vivo* we generally do not observe similar strong expression of HIF target genes as in cell culture *in vitro* (19,36). However, we could not identify any effect on Src signalling following PAG1 induction by hypoxia. Upon phosphorylation by SFKs, PAG1 is known to associate with C-terminal Src kinase (Csk) through Tyr-317, proximal to membrane-associated SFKs (Src, Lck, Hck, Fyn, Blk, Lyn, Fgr, Yes and Yrk). Csk then phosphorylates the C-terminal negative regulatory tyrosine residue of SFKs, which suppresses their activation (37). In our hands, hypoxia neither affected PAG1 tyrosine phosphorylation nor the phosphorylation of any SFK member. This may be attributed to a certain degree of redundancy in PAG1 function because various PAG1-deficient mouse models did not reveal any obvious phenotype (38–40). It could also be that subtle PAG1-mediated changes in SFK function become apparent only at specific time points after the onset of receptor stimulation-mediated Src signalling. However, cell type-specific functional investigations of Src signalling were beyond the scope of the present work.

Because the *PAG1* promoter region did not confer hypoxic inducibility to a reporter gene, we looked for more distal HREs regulating *PAG1* gene expression. Pan-genomic ChIP-seq studies previously identified frequent distal HREs, including distant intronic, exonic and intergenic regions (7,11). We could identify a single −82 kb HRE that is essential for hypoxic *PAG1 cis*-activation, as shown by TALEN-mediated destruction of the HBS within this HRE. To our knowledge, this is the first time that gene editing has been used to functionally analyze an HRE in human cell lines. Using homologous recombination in mouse ES cells, HREs regulating the *Vegf* and *Epo* genes have been studied previously. Deletion of the HRE located in the promoter of the *VEGF* gene unexpectedly resulted in adult-onset progressive motor neuron degeneration reminiscent of amyotrophic lateral sclerosis, probably due to neuronal hypovascularization (41). Mutation of the 3′ HRE regulating the *Epo* gene revealed that this HRE, in contrast to previous belief, mediates hypoxic *Epo* induction in the liver but not the kidney (42). We recently resolved this finding by suggesting the existence of a conserved −9.2 kb HRE within the distal 5′ kidney-inducible element (13).

In contrast to the frequent distal location of HREs suggested by HIFα ChIP-seq, only very few of these sites have been functionally studied so far. For example, upstream HREs −35 kb and −57 kb from the TSS, respectively, mediate the hypoxic induction of the *SLC2A3* (9) and *IGFBP3* (12) genes. The only gene comprehensively analyzed containing an HRE more distant to the TSS than the −82 kb *PAG1* HRE gene reported herein is the *CCND1* gene (encoding cyclin D1) whose HRE is located −220 kb upstream of the TSS (8). Based on genome-wide association studies to screen for population-based cancer susceptibility loci, this intergenic remote HRE has been identified as a susceptibility locus for renal cell carcinoma, regulating *CCND1* expression via HIF-2 specifically in VHL-defective renal cancer cells. The protective haplotype impairs HIF-2 binding and thus links oxygen-sensing with cell cycle control (8). This newly identified polymorphism did not include the consensus HBS, implying that additional elements within this HRE are required for proper *cis*-regulation of hypoxic gene expression.

Although a very recent study (30) reported that HBSs are accessible in normoxia, prior to HIF stabilization the existence of remote HREs raises several additional questions regarding their physical interaction with the promoter region: (i) what is the dynamics of this interaction and how does it vary between different cell types? (ii) Is it affected by different oxygenation, i.e. differences in HIF protein levels? (iii) How precisely is the HRE locus kept in an open chromatin conformation, especially when it is not residing within a methylation-free CpG island? Using 3C assays in MCF-7 and HeLa cells, we found constitutive oxygen and HIFα independent chromatin interaction between the *PAG1* −82 kb HRE and promoter regions. Such a pre-formed chromatin loop, independent of conditional cell signalling, is in line with a previous report demonstrating that TNF-α-responsive enhancers are in contact with their promoters before signalling (43). In fact, in this genome-wide study pre-existing chromatin looping was suggested to be a strong predictor of gene induction which contributes to cell-type specific regulation of conditional gene expression.

Obviously, there is a need for additional *trans*-acting factors, binding at or near the consensus HBS, to confer constitutive oxygen-independent chromatin looping. TALEN-mediated destruction of the HBS apparently only partially abolished chromatin interaction with the promoter region, suggesting that *trans*-acting factors interacting with both the HBS itself as well as with neighbouring sites are involved. We and others previously demonstrated that the canonical HBS can also be bound by ATF-1, CREB-1 and USF transcription factors (33,44–46). These factors may be involved in preventing CpG methylation of unoccupied HBSs outside of CpG islands under normoxic conditions, which is known to block the interaction with HIF (47,48). Alternatively, they may be involved in epigenetic chromatin modification as well as in chromatin looping. While we did not analyze additional transcription factors that may bind at or near the HBS in the −82 kb *PAG1* HRE, several constitutive local chromatin interactions between the HBS and adjacent regions were detected in the 3C assays. Although the probability to detect such interactions by chance substantially increases with proximity, we consistently observed these local loops in all cell lines containing a functional HRE consensus motif. Notably, genome-wide studies identified many different transcription factors binding in the region surrounding the −82 kb *PAG1* HBS (Supplementary Figure S3), suggesting the existence of a larger cluster of transcription factors binding within the HRE, potentially involved in (i) increased recruitment of HIF-2α over HIF-1α to this locus; (ii) keeping the locus in an open chromatin conformation even in the (normoxic) absence of HIF and (iii) enhancing the stability of the long-range enhancer–promoter interaction.

Collectively, our data provide further evidence that a fully functional HRE is defined by a core HBS motif interacting with HIFs as well as by additional proximal DNA binding motifs interacting with other *trans*-acting factors involved in the (co-)recruitment of transcriptional co-activators (*trans*-activation), local chromatin activity (epigenetic modification) and long-range DNA–DNA interactions (chromatin looping). Considering the striking instantaneous stabilization of HIFα protein upon hypoxic stimulation (49), it is likely of major physiological relevance that HREs remain in an open conformation and pre-contact their respective promoters in order to immediately initiate essential adaptation pathways to survive oxygen restriction.

## SUPPLEMENTARY DATA

Supplementary Data are available at NAR Online.

SUPPLEMENTARY DATA
