# Supplementary material for: Destruction of a distal hypoxia response element abolishes trans-activation of the PAG1 gene mediated by HIF-independent chromatin looping
Source: Nucleic Acids Res. 2015 May 24;43(12):5810–23. doi: 10.1093/nar/gkv506 (PMC4499134; doi:10.1093/nar/gkv506)
Supplement: SUPPLEMENTARY DATA [file supp_43_12_5810__index.html]

Destruction of a distal hypoxia response element abolishes trans-activation of the PAG1 gene mediated by HIF-independent chromatin looping — Destruction of a distal hypoxia response element abolishes trans-activation of the PAG1 gene mediated by HIF-independent chromatin looping — SUPPLEMENTARY DATA 

# Destruction of a distal hypoxia response element abolishes *trans*-activation of the *PAG1* gene mediated by HIF-independent chromatin looping

## SUPPLEMENTARY DATA

- SUPPLEMENTARY DATA
