# Supplementary material for: Destruction of a distal hypoxia response element abolishes trans-activation of the PAG1 gene mediated by HIF-independent chromatin looping
Source: Nucleic Acids Res. 2015 May 24;43(12):5810–23. doi: 10.1093/nar/gkv506 (PMC4499134; doi:10.1093/nar/gkv506)

SUPPLEMENTARY DATA

Supplementary Table 1. PCR primers used for the amplification of the -82 kb *PAG1* HRE region.

Supplementary Table 2. ChIP-qPCR primers.

Supplementary Table 3. 3C primers (position relative to *PAG1* TSS).

Supplementary Table 4. Top 100 genes induced in HeLa cells following exposure to hypoxia (0.2% O_2) for 16 hours. Microarray data are shown as fold change (mean values of biological duplicates).

Supplementary Figure 1. PAG1 is induced in CKD patients and in hypoxic human kidney cell lines. (**A**) Gene array expression data were obtained from microdissected glomeruli and tubulointerstitial compartments from patients with glomerulopathies. Values are fold changes relative to living donors for 3 different probesets. (**B**) PAG1 mRNA levels were validated by RT-qPCR in an independent cohort of microdissected samples. PAG1 mRNA levels are shown relative to 18S rRNA. The number of patients is indicated between brackets. LD, living donors; DN, diabetic nephropathy; RPGN, rapidly progressive glomerulonephritis; FSGS, focal-segmental glomerulosclerosis. PAG1 mRNA (**C** to **E**) and protein levels (**C**) were determined in HK2 (**C**) TK188 (**D**) and TZ1 (**E**) cells following exposure to 0.2% O_2 for 24 hours. PAG1 mRNA levels are shown relative to ribosomal protein L28 mRNA levels, error bars correspond to the SEM.

Supplementary Figure 2. PAG1 phosphorylation is not affected by hypoxia. (**A**) PAG1 protein (left panel) and mRNA (right panel) levels as determined by immunoblotting and RT-qPCR following stable transfection of Hep3B cells with shPAG1 or shCtrl. PAG1 mRNA levels are shown relative to ribosomal protein L28 mRNA levels, error bars correspond to the SEM. (**B**) Human Phospho-Kinase array incubation with lysates of shCtrl or shPAG1 Hep3B cells following exposure to 20% O_2 or 0.2% O_2 for 24 hours. Dark dots on the upper and lower corner of the membranes represent undisclosed positive controls. The rectangles indicate the following SFKs: Src, Lyn, Fyn and Yes (duplicates; clockwise from upper left). (**C**) PAG1 tyrosine phosphorylation in Hep3B cells exposed to 20% O_2 or 0.2% O_2 for 24 hours. Anti-PAG1 or IgG control antibodies were used for Immunoprecipitation, followed by immunoblotting and detection with a pan-phosphotyrosine antibody.

Supplementary Figure 3. ChIP-sequencing reads covering the -82 kb *PAG1* HRE. UCSC Genome Browser output *(hg19)* indicating the HIF ChIP-sequencing reads for HIF-1α, HIF-2α and HIFβ in 786-0 and MCF-7 cells. Below a closer view is shown of the 82 kb *PAG1* HRE surrounding region with ENCODE-integrated DNase hypersensitivity clusters, histone marks and transcription factor occupancy.

Supplementary Figure 4. Destruction of the -82 kb *PAG1* HRE abolishes hypoxic PAG1 mRNA induction but not CAIX mRNA or HIFα protein induction. (**A**) Overview of the two pairs of TALEN vectors (TPI and TPII) used to target the -82 kb *PAG1* HRE. The core HBS and FokI cleavage sites are indicated. (**B**) Strategy to detect TALEN-mediated destruction of the HBS within the -82 kb *PAG1* HRE following NHEJ DNA repair. (**C**) Genotyping of representative targeted HeLa (left panel) and MCF-7 (right panel) clones by PCR amplification of the -82 kb *PAG1* HRE region followed by BsaAI restriction digestion and agarose gel electrophoresis. Following exposure of HeLa (**D**, **F**) and MCF-7 (**C**, **G**) clones to 20% O_2 or 0.2% O_2 for 24 hours, PAG1 and CAIX mRNA levels (**D**, **E**) as well as HIF-2α, HIF-1α, PAG1 and β-actin protein levels (**F**, **G**) were determined by RT-qPCR and immunoblotting, respectively. Transcript levels are shown relative to β-actin mRNA levels (n = 3), error bars correspond to the SEM. Statistical analyses were performed with unpaired Student's t-tests (*, $P<0.05$; **, $P<0.01$; ***, $P<0.001$).

Supplementary Table 1

Primer	Sequence (5'-3')
317 bp fwd	TGGAAGATCTTGCCAATAATAGCATGCTGG
317 bp rev	TGGAAGATCTTTACAATAATTAGAGGCTTT
Enh_2 kb fwd	TGGAAGATCTTTACCTCCCAGTTGTCTGAAC
Enh_2 kb rev	TGGAAGATCTCTTGTGCCAAATCAGGCTAT

Supplementary Table 2

ChIP-qPCR primer	Primer fwd Sequence (5'-3')	Primer rev Sequence (5'-3')
β-actin	ACCATGGATGATGATATCGCC	GCCTTGCACATGCCGG
EGLN3	AGTGTCCGTTCCCAGCTCAG	TAGGCACAGTAAACAGGCC
PAG1 enhancer	TGCCAATAATAGCATGCTGG	TACAATAATTAGAGGCTTT

Supplementary Table 3

3C primer	Sequence (5'-3')
Anchor (-83637)	AAACACAGTAAATTCTCCAAAGCAG
7631	AACAACAATTTCAAATACACGGAAG
1107	TGAAATCCAAGTGTCATTATCTGAA
-665	ATCCTTGCTACTTAACGTGTGATCT
-2216	TCTAACTCTTCCATTACAGCCATTC
-2700	CAGCTACATCTTTTTCTCTGAGGTC
-5198	TTTTCTGAAAGAATCAGCCATTTAC
-17851	TACAAAATTGTCCTTCAGAAGACCT
-80655	TCACGTAAACCTTCAGTTTTCCTAC
-81310	CCATGTTAACCAAAGAGGTATCATC
-84203	TAATCTACCCCTTGTTTAGCATGTC
-86938	GCTTAATTTTAGATGAGAAGGCTCAA

Supplementary Table 4

Number	Gene	Fold change	Number	Gene	Fold change
1	CA9	74.76	51	SFXN3	3.1
2	PPFIA4	28.33	52	GOLGA8A	3.092
3	PFKFB4	18.62	53	KCTD11	3.053
4	NDRG1	18.38	54	HEY1	3.051
5	ANGPTL4	15.22	55	JMJD1A	3.022
6	SPAG4	11.89	56	MAFF	3.01
7	IGFBP3	8.351	57	HK2	2.968
8	EGLN3	7.464	58	B3GNT4	2.966
9	LOX	7.25	59	WDR54	2.957
10	ITGB4	7.16	60	DDR1	2.94
11	ERRFI1	6.346	**61**	**PAG1**	**2.932**
12	STC1	6.193	62	SFXN3	2.907
13	ADM	6.12	63	CXCR4	2.881
14	BNIP3L	5.845	64	FOS	2.854
15	SLC2A3	5.56	65	RNASE4	2.812
16	INSIG2	5.532	66	HILPDA	2.798
17	LOXL2	5.307	67	SH3PX3	2.787
18	SLC2A14	5.253	68	PPP1R3E	2.787
19	TMEM45A	5.201	69	ERRFI1	2.771
20	DLX4	4.92	70	GOLGA8A	2.767
21	ALDOC	4.896	71	RAB40C	2.76
22	AK3L1	4.768	72	AK3L1	2.708
23	CCNG2	4.673	73	PLOD2	2.706
24	CRABP2	4.502	74	DDIT4	2.674
25	MYO7B	4.271	75	GOLGA9P	2.674
26	PPP1R3B	4.172	76	SPTLC2L	2.649
27	ADFP	4.115	77	TPBG	2.648
28	MUC1	4.053	78	ANO7	2.647
29	ANKRD37	4.047	79	TMPRSS3	2.583
30	GPR146	3.992	80	TIMM13	2.573
31	KIAA1199	3.86	81	ARID5A	2.561
32	S1PR4	3.792	82	HK1	2.556
33	BHLHB2	3.733	83	TTYH3	2.555
34	FAM13A1	3.577	84	FAM162A	2.523
35	IER3	3.5	85	ATP1B1	2.515
36	VLDLR	3.5	86	OSMR	2.506
37	ITGA5	3.462	87	GBE1	2.5
38	ARRDC3	3.456	88	FAM83A	2.483
39	PPP2R5B	3.423	89	FAM139A	2.476
40	PDK1	3.374	90	BTG1	2.464
41	ANKZF1	3.354	91	CNOT8	2.441
42	PDK1	3.354	92	SLC6A6	2.433
43	TMPRSS3	3.353	93	PGK1	2.408
44	MXI1	3.304	94	FAM57A	2.387
45	AK3L1	3.301	95	GPRC5A	2.357
46	BHLHB2	3.252	96	C8orf58	2.345
47	TMEM145	3.24	97	FOXN4	2.335
48	ANG	3.216	98	QSOX1	2.327
49	ZNF395	3.15	99	ITGB8	2.325
50	WFIKKN1	3.121	100	RRAGA	2.317

Supplementary Figure 1

A

	Probe ID	DN	FSGS	RPGN
Glomeruli	227354_at	2.061	1.769	1.941
	225626_at	2.116	2.229	1.948
	225622_at	1.838	1.687	1.660
Tubulo-interstitium	227354_at	1.430		1.452
	225626_at	1.393		1.299
	225622_at			1.460

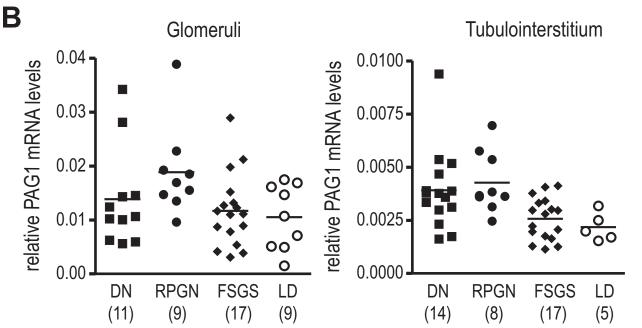

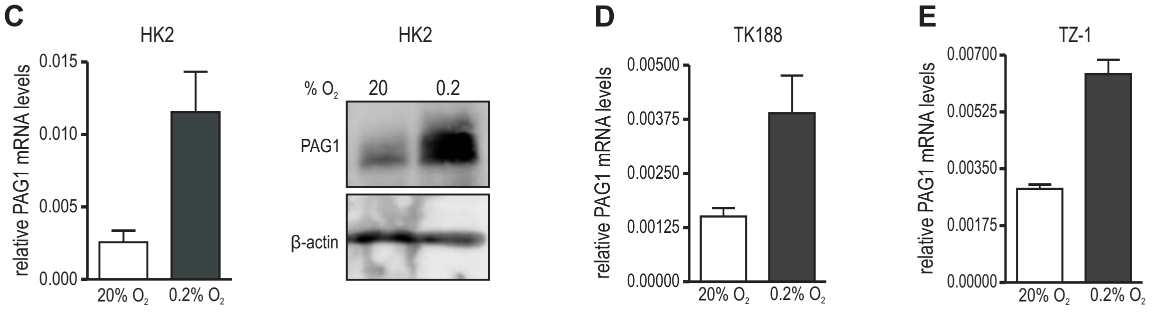

Supplementary Figure 2

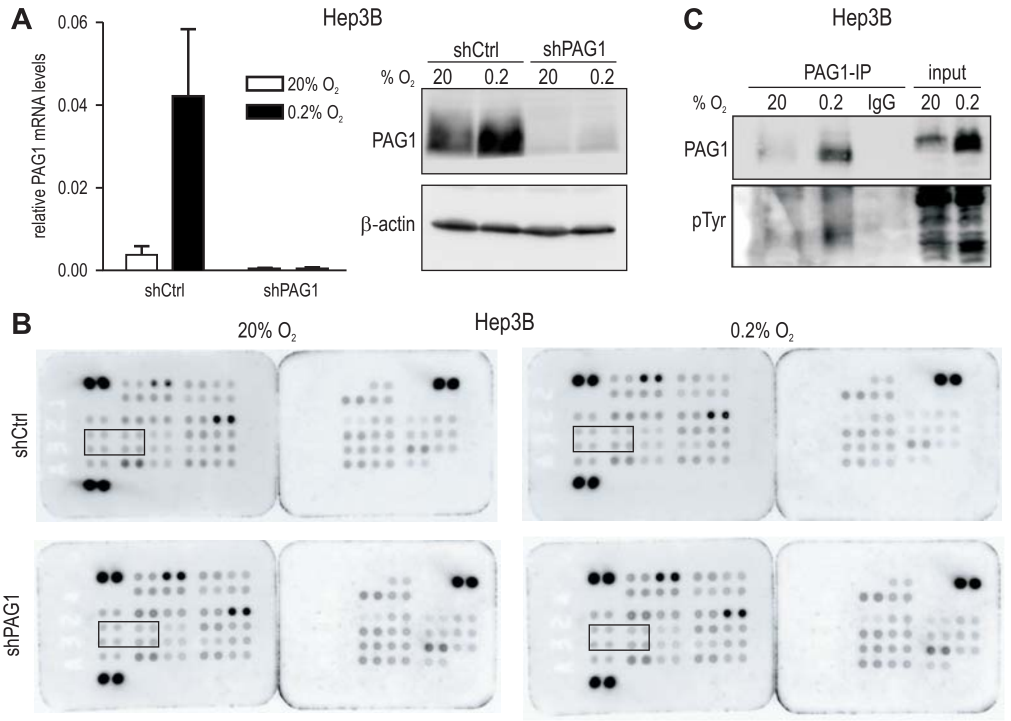

Supplementary Figure 3

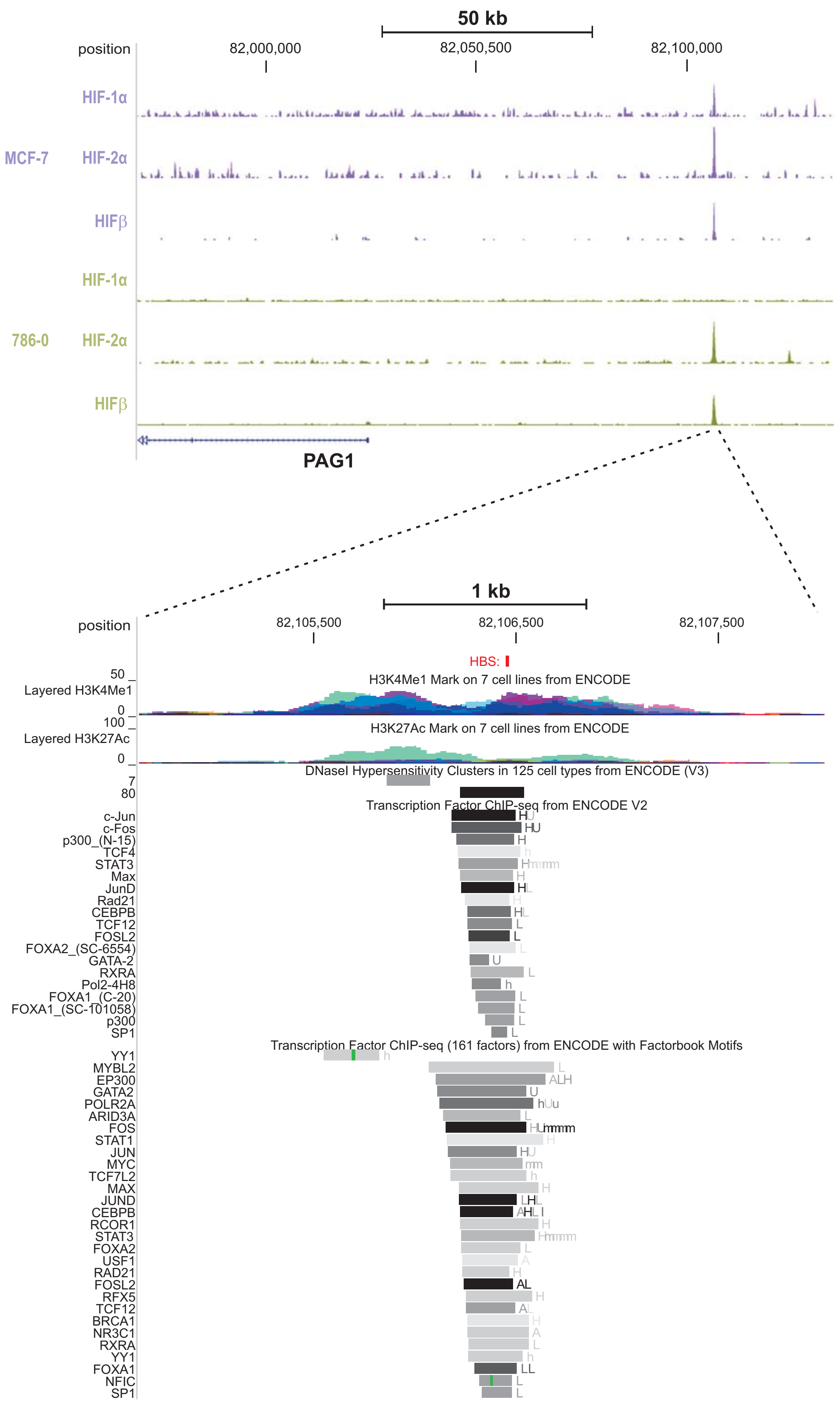

Schörg, Santambrogio et al., Supplementary Figure 4

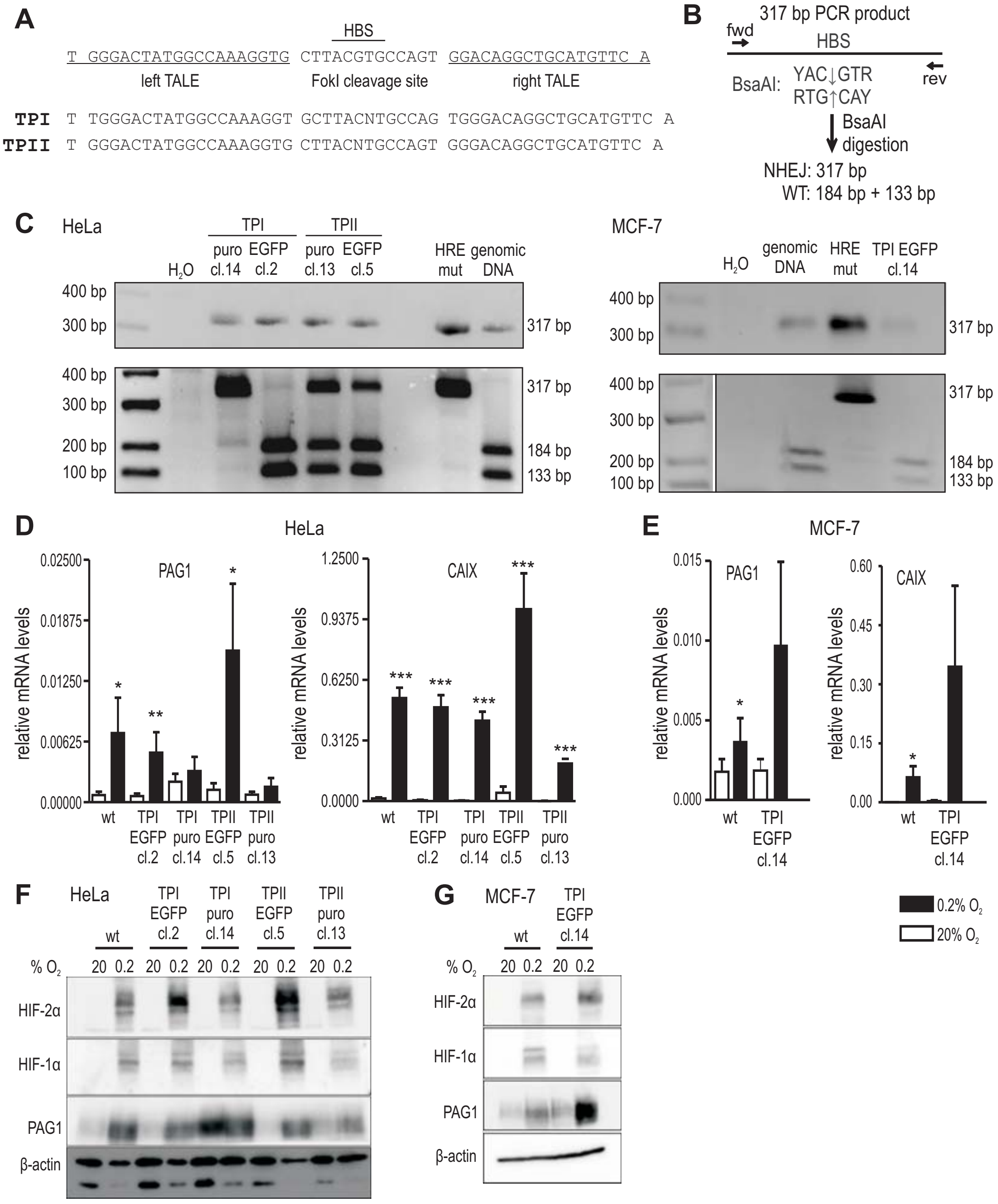

Supplement: SUPPLEMENTARY DATA [file supp_gkv506_nar-01139-v-2015-File008.pdf]
